# An Italian Study of PM_0.5_ Toxicity: In Vitro Investigation of Cytotoxicity, Oxidative Stress, Intercellular Communication, and Extracellular Matrix Metalloproteases

**DOI:** 10.3390/ijms26146769

**Published:** 2025-07-15

**Authors:** Nathalie Steimberg, Giovanna Mazzoleni, Jennifer Boniotti, Milena Villarini, Massimo Moretti, Annalaura Carducci, Marco Verani, Tiziana Grassi, Francesca Serio, Sara Bonetta, Elisabetta Carraro, Alberto Bonetti, Silvia Bonizzoni, Umberto Gelatti, the MAPEC_LIFE Study Group

**Affiliations:** 1 Laboratory of Tissue Engineering, Department of Clinical and Experimental Sciences, University of Brescia, Viale Europa, 11, 25123 Brescia, Italy; giovanna.mazzoleni@unibs.it (G.M.); jennifer.boniotti@unibs.it (J.B.); 2 Research Center “Integrated Models for Prevention and Protection in Environmental and Occupational Health” (MISTRAL), Department of Medical and Surgical Specialties, Radiological Sciences, and Public Health, University of Brescia, 25123 Brescia, Italy; 3 Department of Pharmaceutical Sciences, University of Perugia, 06122 Perugia, Italy; milena.villarini@unipg.it (M.V.); massimo.moretti@unipg.it (M.M.); 4 Department of Biology, University of Pisa, 56127 Pisa, Italy; annalaura.carducci@unipi.it (A.C.); marco.verani@unipi.it (M.V.); 5 Department of Experimental Medicine, University of Salento, 73100 Lecce, Italy; tiziana.grassi@unisalento.it; 6 Department of Biological and Environmental Sciences and Technologies, University of Salento, 73100 Lecce, Italy; francesca.serio@unisalento.it; 7 Department of Public Health and Pediatrics, University of Turin, 10126 Torino, Italy; sara.bonetta@unito.it (S.B.); elisabetta.carraro@unito.it (E.C.); 8 Multisectoral and Technological Services Centre (CSMT) Innovation Hub, 25123 Brescia, Italy; a.bonetti@csmt.it; 9 Comune di Brescia, 25123 Brescia, Italy; 10 Department of Medical and Surgical Specialities, Radiological Sciences and Public Health, University of Brescia, 25123 Brescia, Italy; umberto.gelatti@unibs.it

**Keywords:** Italian airborne PM_0.5_, in vitro cytotoxicity, cell growth, tumour promotion, oxidative stress, MMP activity

## Abstract

Particulate matter (PM), mainly PM_0.5_, represents a significant concern for human health, particularly relating to lung homeostasis, and more research is required to ascertain its tissue tropism and the molecular pathways involved. In this study, we first focus on classical in vitro toxicological endpoints (cytotoxicity and cell growth) in human bronchial and alveolar epithelial cell lines mimicking the two pulmonary target tissues. Air samples were collected in five Italian cities (Brescia, Lecce, Perugia, Pisa, Turin) during winter and spring. To better decipher the PM_0.5_ effects on pulmonary cells, a further winter sampling was performed in Brescia, and studies were extended to assess tumour promotion, oxidative stress, and the activity of Matrix metalloproteases (MMP). The results confirmed that the effect of air pollution is linked to the seasons (winter is usually more cytotoxic than spring) and is correlated with the peculiar characteristics of the cities studied (meteoclimatic conditions, economic/anthropogenic activities). Alveolar cells were often less sensitive than bronchial cells. All PM samples from Brescia inhibited intercellular communication mediated by gap junctions (GJIC), increased the total content in glutathione, and decreased the reduced form of glutathione, whereas the Reactive Oxygen Species (ROS) content was almost constant. Long-term treatments at higher doses of PM decreased MMP2 and MMP9 activity. Taken together, the results confirmed that PM is cytotoxic and can potentially act as tumour promoters, but the mechanisms involved in oxidative stress and lung homeostasis are dose- and time-dependent and quite complex.

## 1. Introduction

In recent decades, the scientific literature has shown that air pollution is an important risk factor for human health, both in adults and in the paediatric population, and a major cause of morbidity and mortality [1,2,3]. Although particulate matter can harm various systems and organs, the main pathologies concern pulmonary (deficit in pulmonary functionality, stunted lung development in children, airway inflammation, asthma, pain, dyspnoea, and coughing) and cardiovascular (ischemia, arrhythmia, and hypertension) systems [4,5,6,7,8]. World Health Organization (WHO) studies showed that about 7 million deaths were related to ambient air pollution, contributing to 7.6% of all deaths [9,10]. Regarding air quality, despite international agreements and internal health policies leading to reduced air pollutant emissions in recent years, atmospheric pollution remains a considerable risk to humans [11,12]. It has been highlighted that children are more vulnerable to air pollution than adults for several reasons: children’s greater physical activity and exposure to the open air, the anatomical and functional immaturity of some of their organs, and the greater quantity of inhaled air per unit of body weight [13,14,15]. From this, interest has arisen in evaluating the effects of air pollution on the paediatric population to identify population-level predictors of biological damage caused by the onset of chronic diseases in adulthood.

PM may have an impact on pulmonary homeostasis by disrupting the oxidant/antioxidant balance, inducing inflammation and related pathologies [16]. Based on our expertise, this study, as part of the MAPEC_LIFE project, thus aimed to investigate the in vitro “baseline” toxicity (cell growth and cell survival) of ultra-fine PM collected in five different Italian cities (Brescia, Turin, Pisa, Perugia, and Lecce) in two seasons (winter I and spring I). The project’s results thus far have encouraged us to expand our research into seeking more specific targets of PM-derived toxicity, using samples from Brescia collected in three independent seasons (winter I, spring I, winter II). Molecular targets such as tumour promotion (GJIC), oxidative stress (ROS and Glutathion (GSH)), and MMPs (enzymes involved in lung tissue homeostasis and repair) were evaluated because these pathways are often influenced by particulate matter and in response to agents with pro-inflammatory and carcinogenic potential [17,18]. We believed that adopting this “multi-test” approach to investigate the toxic potential of PM0.5 could provide more detailed information than the “traditional” in vitro toxicology strategy, which is only based on cell viability assessment via MTT((3-(4,5-dimetiltiazol-2-il)-2,5-difeniltetrazolio)/RN (Neutral Red Uptake) tests, for example.

## 2. Results

### 2.1. Baseline Toxicity Assessment

#### 2.1.1. Impact of PM on Cell Growth

**Figure 1:**
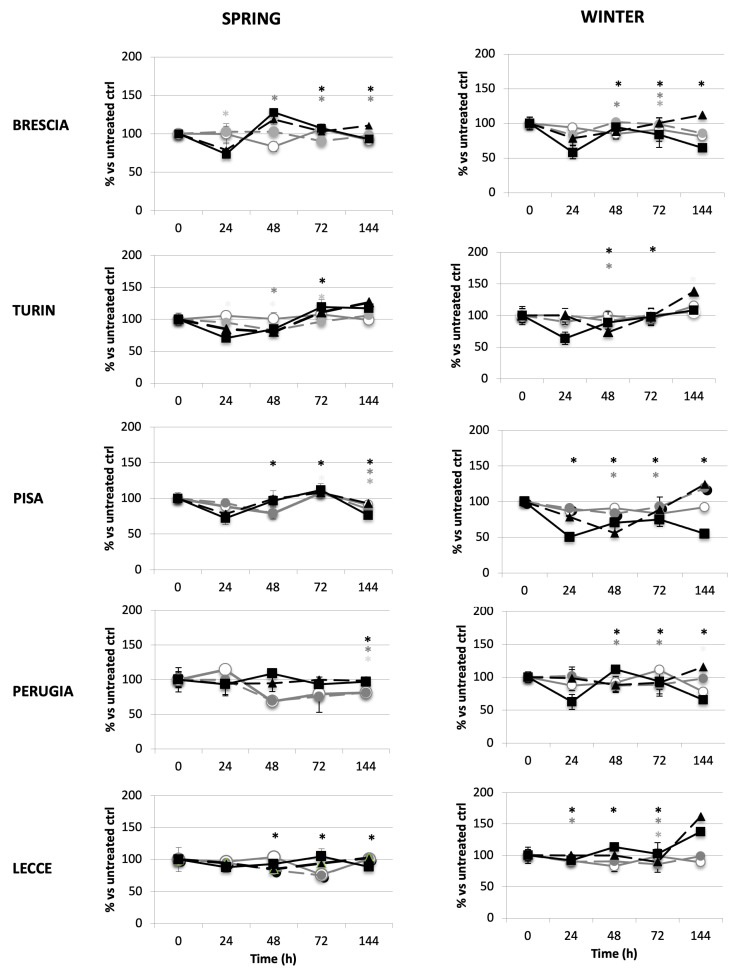
Effect of air samples on bronchial cell growth. BEAS-2B bronchial cells were treated with 1 m^3^Eq (grey continuous line and white circle), 2.5 m^3^Eq (grey broken line and grey circle), 5 m^3^Eq (black broken line and black triangle), and 7.5 m^3^Eq (black continuous line and black square) of air samples originating from Brescia, Turin, Pisa, Perugia, and Lecce and were maintained in culture up to 144 h. High-volume air samplers for atmospheric particulates have been used to withdraw and concentrate ultra-fine particulate samples (PM_0.5_), which were concentrated on glass fibreglass filters, and after gravimetric determination of PM_0.5_ mass, they were processed with organic extraction. A unique pool of collected samples from each city was tested (see Section 4 for period of sample collection). Cells were automatically counted (TC20, Biorad, Italy). Results are expressed as percentages versus the untreated control. Experiments were performed in triplicate dishes. (*p* < 0.05 for 1 m^3^Eq*, 2.5 m^3^Eq *, 5 m^3^Eq *, 7.5 m^3^Eq *).

Cell growth was differently affected by the treatment with samples extracted from the diverse cities (Figure 1). Concerning BEAS cells, samples collected at Lecce induced less cytotoxicity than the others, whereas the Pisa and Brescia samples exhibited the highest toxicity. For the higher doses, a biphasic effect was often observed; after 24/48 h of treatment, the cell growth was decreased, whereas, for a longer culture time, the growth roughly reached the level of the untreated control. An evident difference in cytotoxicity can be observed between winter and spring, such that for almost all cities winter is the season with higher toxicity, exhibiting up to 50% growth inhibition; however, surprisingly the spring air in Lecce was more toxic than the winter air.

**Figure 2:**
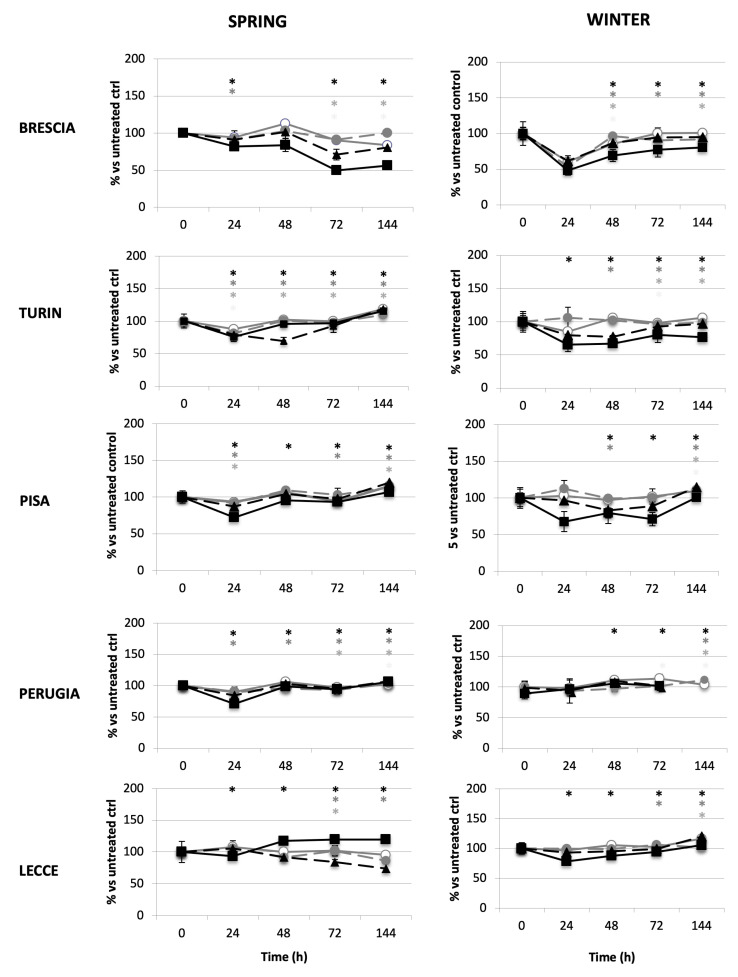
Effect of air samples on alveolar cell growth. A549 alveolar cells were treated with 1 m^3^Eq (grey continuous line and white circle), 2.5 m^3^Eq (grey broken line and grey circle), 5 m^3^Eq (black broken line and black triangle), and 7.5 m^3^Eq (black continuous line and black square) of air samples originating from Brescia, Turin, Pisa, Perugia, and Lecce and were maintained in culture up to 144 h. Cells were automatically counted (TC20, Biorad). High-volume air samplers for atmospheric particulates have been used to withdraw and concentrate ultra-fine particulate samples (PM_0.5_), which were concentrated on glass fibreglass filters, and after gravimetric determination of PM_0.5_ mass, they were processed with organic extraction. A unique pool of collected samples from each city was tested (see Section 4 for period of sample collection). Cells were automatically counted (TC20, Biorad, Italy). Results are expressed as percentages versus the untreated control. Experiments were performed in triplicate dishes. (*p* < 0.05 for 1 m^3^Eq*, 2.5 m^3^Eq *, 5 m^3^Eq *, 7.5 m^3^Eq *).

The samples originating from Pisa, Brescia, and Turin highly modified the growth of the A549 alveolar cells (Figure 2).

Among all the winter samples, Brescia is unique in reaching IC50 (i.e., inhibition of 50% cell viability, after 24 h treatment with 7.5 m^3^Eq). Lecce- and Perugia-derived samples present a lower toxic effect on alveolar cells. Except for Perugia, spring samples are less toxic than winter ones. Moreover, samples originating from Lecce in spring present long-term cytotoxicity for the lower doses, whereas at the higher concentration (7.5 m^3^Eq), the early modest toxicity is quickly reversed. For Brescia and Turin, the highest toxicity observed with the higher dose is hardly reversed. In addition, alveolar and bronchial cells do not present the same sensitivity to air components. Except for Turin- and Brescia-derived samples, bronchial cells are almost always more sensitive than alveolar cells.

#### 2.1.2. Impact of PM on Cell Viability

**Figure 3:**
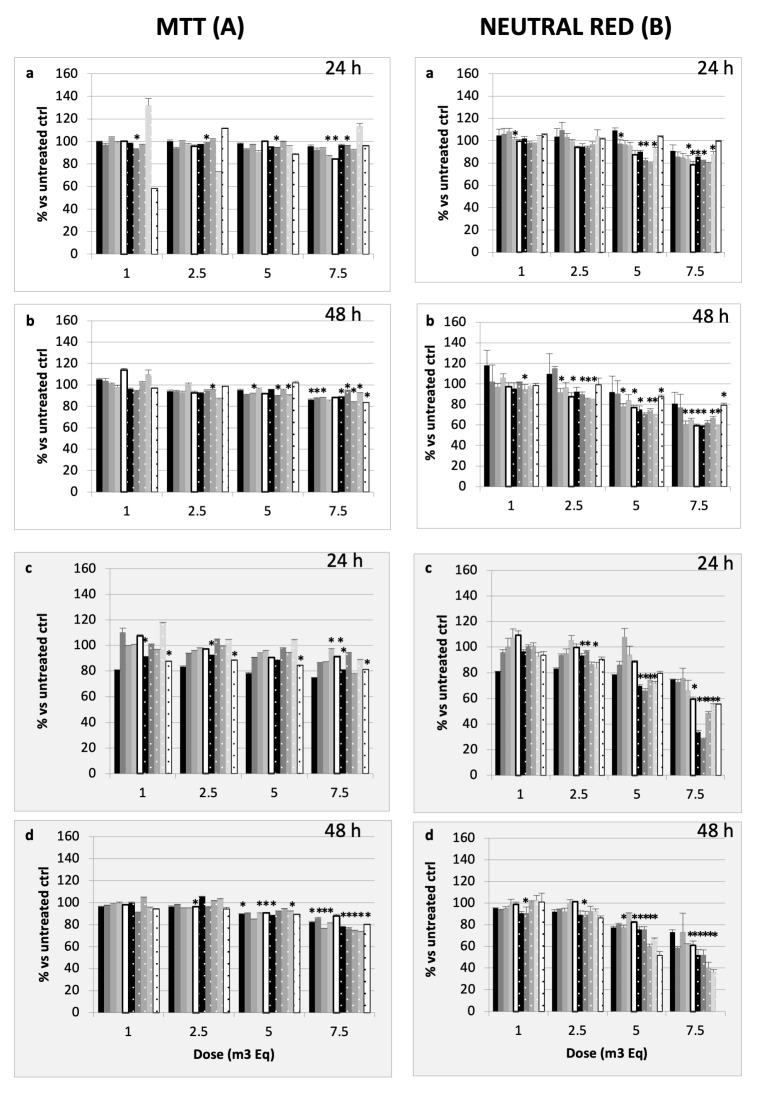
Alveolar and bronchial cell viability was assessed with MTT (**A**) or neutral red (**B**) assays. Experiments were performed in triplicate wells; cells (A549—white background (**a**,**b**); BEAS-2B cells—grey background (**c**,**d**)) were treated for 24 h (**a**,**c**) or 48 h (**b**,**d**) with increasing doses of PM_0.5_. Experimental conditions can be found in the Section 4. Results are expressed as a percentage vs. untreated control ± SD. Solid colours represent the spring season: Black: BS; Grey: TO, Intermediate grey; Pisa, Light grey: Perugia; White: Lecce. Note: the winter season is white dotted: Black: BS; Grey: TO; Intermediate grey: Pisa; Light grey: Perugia; White: Lecce. * = *p* < 0.05.

A neutral red assay performed on the alveolar epithelial cells showed that, except for Lecce, the 24 h treatment with 7.5 m^3^ induced a decrease in the cell count up to 20% (Figure 3(Aa)), whereas it reached 40% at 48 h (Figure 3(Ab)). The MTT test (Figure 3(Ba,Bb)) confirmed that cell metabolism was not critically inhibited by the various treatments. Concerning the bronchial cells, the results confirmed that after either 24 h or 48 h, the main toxic effect was observed for the higher doses tested (5 and 7.5 m^3^Eq) and that samples collected in winter were significantly more cytotoxic than those in spring (Figure 3(Ac,Ad)). This decrease is more evident than that observed with alveolar epithelial cells. Air samples from Brescia, Turin, and Pisa were often more cytotoxic than samples from the other cities. After 48 h of treatment (Figure 3), the results were similar, even if the cell viability was reduced more than at 24 h. For the highest dose (7.5 m^3^Eq), winter resulted in more toxicity for all cities. The results confirmed the trend observed the cell growth. On the other hand, the MTT test (Figure 3(Bc,Bd)) showed that the cell metabolic activity was poorly inhibited by the various samples. The cell count was never reduced by more than 20%, regardless of the city of origin. Moreover, the results confirmed that bronchial cells are more sensitive to organic compounds than alveolar cells.

### 2.2. Specific Targets of PM-Derived Toxicity: Focusing on Brescia’s Airborne Particles

#### 2.2.1. Cell Viability Assessment

**Figure 4:**
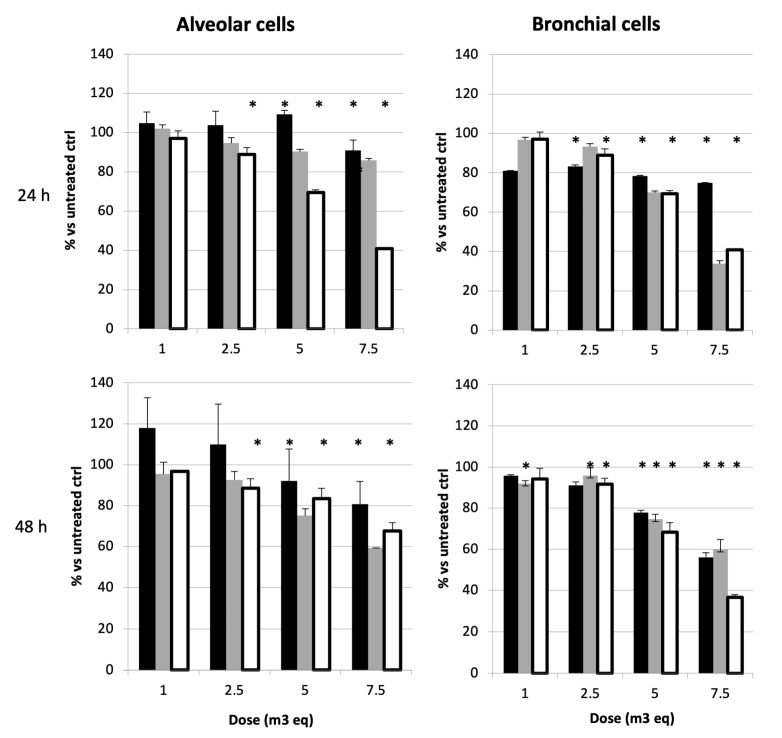
Effect of Brescia-derived samples on the alveolar and bronchial cell viability. Cell viability was assessed via the neutral red assay. The detailed experimental conditions are described in Section 4. Cells (A549 or BEAS-2B cells) were treated for 24 h and 48 h with winter I (black), spring (grey), and winter II (white) samples derived from Brescia in triplicate wells. Results are expressed as a percentage of viability of treated cells vs. untreated control ± SD. * = *p* < 0.05.

In our study, Brescia represents one of the most polluted cities, and the neutral red assay was extended to another winter season (winter II). The results are presented in Figure 4 and show that the highest dose, 7.5 m^3^Eq, is highly toxic for both alveolar and bronchial cells. In addition, it is evident that spring-derived samples never induced a 50% decrease in cell viability, whereas winters I and II both induced critical cell death at 24 h at doses between 6.1 and 6.7 m^3^Eq for both cell types (Table 1). Determining the IC50 after 24 h and 48 h treatments confirms that bronchial cells are often more sensitive than alveolar cells. The highest cytotoxic activities were observed at 24 h, and except for winter II in bronchial cells (IC_50_ = 6.3 m^3^Eq), the IC50 was not determinable at 48 h.

#### 2.2.2. Tumour Promotion Evaluation

Because the Brescia air samples showed critical side effects, we focused our attention on more defined endpoints for the city. First, we studied the tumour promoter potential of the air compounds. Because BEAS-2B cells did not have sufficient gap junctions, they were not included in this study.

A scrape loading assay was performed on alveolar epithelial cells (A549) and hepatic cells (IAR203), which communicate well. A 6 h treatment at the 1 m^3^Eq dose was chosen because regardless of the sample considered, the 20% inhibitor dose was always out of range, whereas at 2.5 m^3^Eq, some samples were already too toxic to perform such an investigation.

**Figure 5:**
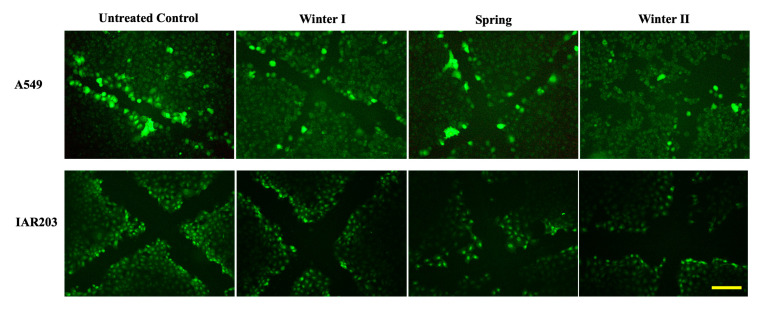
Effect of air pollutants on intercellular communication mediated by gap junctions (GJIC): assessment of tumour promotion potential. The scrape loading method was applied to A549 alveolar cells or IAR203 hepatic epithelial cells treated for six hours with the 1 m^3^Eq dose. Image analysis was performed on PFA-fixed cells observed under fluorescence microscopy (Olympus) and using the CellSens 4.1 software program (Olympus). Photographs show the extension of the Lucifer Yellow staining from damaged to healthy cells (in untreated cells, thus communication was mediated by the gap junctions) and the lack of extension of the fluorescent Lucifer Yellow between A549 cells or reduced communication (in IAR203). Experiments were performed at minimum in duplicate in at least two independent experiments. Bar: 40 μm.

As shown in Figure 5, whatever the season tested, treatments induced substantial inhibition of intercellular communication mediated by gap junctions. Inhibition was higher with samples collected in winter II compared with winter I. The effect was more evident in hepatic cells as they essentially communicate more than alveolar cells (Table 2).

#### 2.2.3. Oxidative Stress Analysis

**Figure 6:**
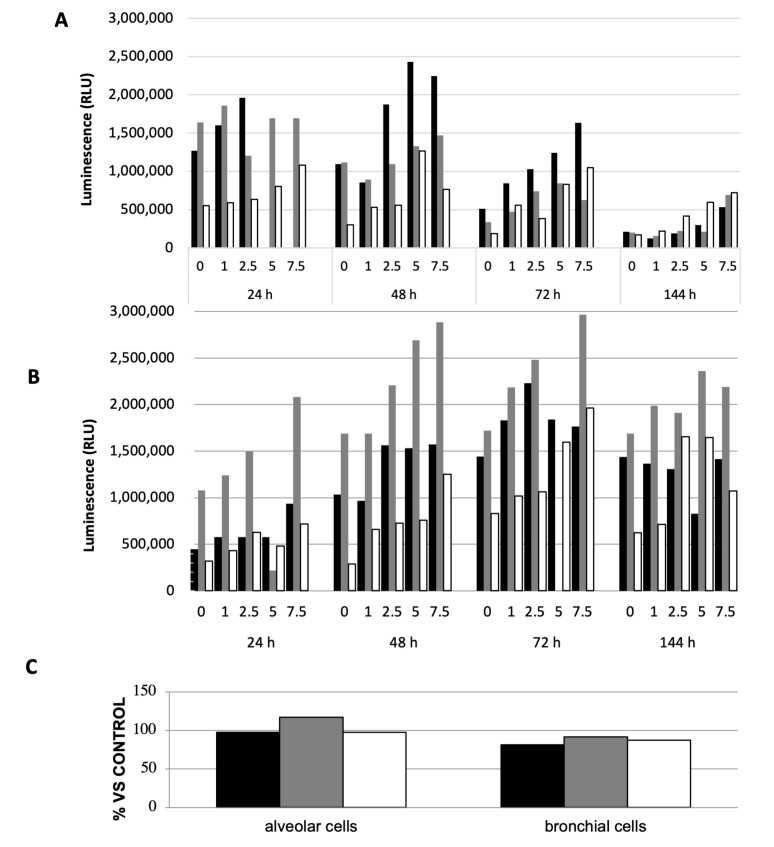
Redox status. GSH and GSSG forms of glutathione (**A**,**B**) and ROS (**C**) were quantified in alveolar cells (**A**) and bronchial (**B**) cells. Experiments were performed with winter I (black), spring (grey) and winter II (white) samples from Brescia. (**C**): Quantification of ROS in Alveolar and Bronchial cells treated with the 1 m^3^Eq for six hours. The detailed experimental conditions are described in the Section 4. Results are expressed as RLU (Relative Light Units) or percentage vs. untreated control. Experiments were performed in singlicate with Promega kits as described by the manufacturer.

Afterwards, we focused our attention on some stress oxidative markers. The glutathione content was determined in alveolar and bronchial cells (Figure 6).

Alveolar cells exposed to increasing doses of air samples presented a decreasing content of reduced glutathione. However, the spring samples had a smaller impact on the glutathione content than the two winter-derived samples. A noteworthy lowering in total glutathione content can be observed for alveolar cells with the cumulative culture time, independently of any treatment. More important is the decrease in total glutathione, the more the oxidised form is prevalent. Contrary to what was observed for alveolar cells, the total glutathione content increased during the whole culture time in bronchial cells. The highest dose of treatment almost always induced the total oxidation of glutathione. In contrast with the glutathione content, ROS levels were poorly modified by the 6 h treatment with 1 m^3^Eq (Figure 6). For the alveolar cells, a slight increase in ROS was observed with the spring-derived samples. The ROS levels in the bronchial cells were always lower than those in the untreated control.

#### 2.2.4. Lung Microenvironment Modulation

**Figure 7:**
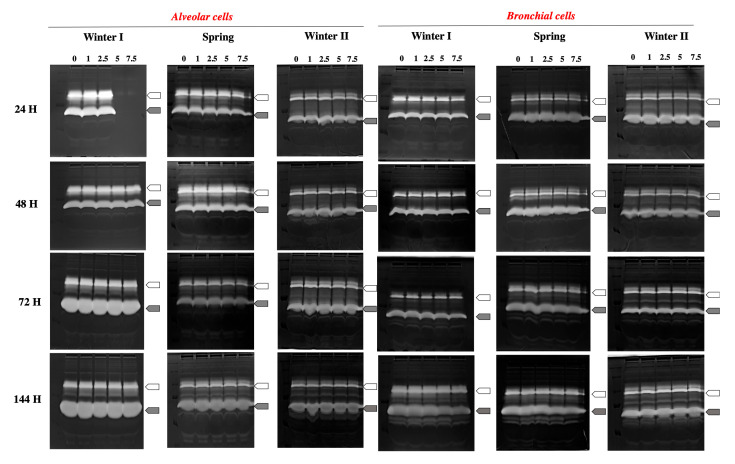
Evaluation of metalloprotease activity in airway-originating cells exposed to air samples originating from Brescia during winter I, spring, and winter II seasons. Culture media harvested from alveolar and bronchial epithelial cells exposed to variable doses of 1, 2.5, 5, and 7.5 m^3^Eq up to 144 h were studied via zymography. An amount of 50 μL of the sample was loaded into each well of the acrylamide/bisacrylamide gel containing gelatin. After electrophoretic migration, gels were incubated at 37 °C for 48 h and stained for 30 min with Coomassie Blue. MMP9 (white arrow) and MMP2 (grey arrow) were highly expressed in airway cells. Experiments were performed in singulate.

Because MMPs are involved in lung homeostasis, the MMP activity was studied throughout the culture time (from 24 to 144 h). Alveolar cells presented a higher basal level of MMP activity than bronchial cells, and as for BEAS-2B cells, it increased with the culture time (see control variations). The treatment with 7.5 m^3^Eq mostly induced a decrease in MMP activity for both cell types (Figure 7, Table 3). The MMP activity trend did not vary between seasons, and the MMP isoforms are differentially regulated. The decrease in MMP activity mainly concerned the activated form of MMPs, but the ProMMP isoforms are also disturbed at the highest dose (7.5 m^3^). In bronchial cells, MMP2 activity is mostly more inhibited than MMP9 activity. Moreover, these cells are less sensitive to treatment with outdoor air.

## 3. Discussion

Italy is one of the most polluted countries in the EU. To explore this aspect, we studied the impact of atmospheric particulates in five cities located in various parts of Italy on cell growth and viability, keeping in mind the main known factors that can influence air quality. The cities of Brescia, Lecce, Perugia, Pisa, and Turin were thus chosen; each of these cities is characterised by its geographical location, topography, population density, pollution level, etc. Moreover, having recognised the impact of the “season” factor on the risk assessment, the samplings were performed during winter and spring. Furthermore, for Brescia, one last winter II sampling was added to evaluate potential variations between winter seasons.

Cell growth was differently affected by air samples according to their provenance. Cities from the North of Italy, such as Brescia and Turin, presented a more cytotoxic pattern, which could be explained by the higher concentrations of PM_10_ quantified in these two cities [19]. A reduction in pulmonary cell growth (alveolar or bronchial cells) in response to PM_10_ and PM_2.5_ has already been documented [20,21]. In these two cities, the anthropogenic activities/impacts on the environment are substantial. Turin and Brescia represent the most populated cities, with the highest number of circulating cars, domestic heating, and most importantly industrial activity. Moreover, in the Po Valley, the freight transport using the roads greatly impacts the environment. In addition, these characteristics represent the main sources of PM emissions (house heating, wood burning, car exhaust, industrial emissions). Furthermore, Turin and Brescia are located in the Po valley, a large plain with peculiar weather characteristics (lowest wind speed, highest humidity, and lowest winter temperatures) that could also contribute toward favouring the accumulation of PM and the condensation of atmospheric pollutants in the particle phase [22]. Other authors have already described the high concentration of atmospheric pollutants in the North of Italy [23,24,25,26]. Brescia and Turin also together have a higher number of circulating cars as compared to Lecce, Perugia, and Pisa, and even if car parks are a more recent addition in Turin and Brescia, the higher number of transiting cars negatively affects air quality. Despite cities developing alternative transport strategies (public transport, bicycle, sharing mobility, etc.), which are qualitatively lacking in Italy as compared to other European countries, a positive impact has not been reached. The minor air cytotoxicity levels in Lecce and Perugia can be explained by the minor concentrations of PM_10_ (15 and 23 μg/m^3^, respectively) as compared to cities with higher levels of toxicity, namely Brescia and Turin (40 and 62 μg/m^3^, respectively) [19]. Concerning Pisa, despite its geographical location, urban topography, and weather seeming more favourable than those of the North-East zone (Turin, Brescia), the local production of PM is significant (13.7 μg/m^3^), being mainly due to transport (over 60% according to the municipality of Pisa). The seasonal variations in atmospheric pollutant concentrations, i.e., spring vs. winter, are thought to be linked, relating not only to the suspension of home heating during spring but also to the meteorological context. Bonetta et al. showed that pollutant concentrations were reduced in almost all samples during springtime. PM_0.5_ variations ranged from #22.8 to 8.2 μg/m^3^; #12.6 to 11.8 μg/m^3^; #13.7 to 5.8 μg/m^3^; #9.9 to 5.6 μg/m^3^; and #7.1 to 4.4 μg/m^3^ for Turin, Brescia, Pisa, Perugia, and Lecce, respectively. Whereas the PM_0.5_ concentration decreased during spring, Lecce’s samples were slightly more toxic than during winter. Despite the winter presenting more rainy weather than the spring, which should have led to lower PM concentrations, the winter PM_0.5_ concentration remained higher than in spring [27]. These results suggest that toxicity is not only a result of PM_0.5_ concentration but also PM composition. Concerning Brescia, the constant PM_0.5_ concentration between winter I and spring (12.6 and 11.8 μg/m^3^, respectively) observed by Bonetta and coworkers [19] could be explained by the warmer winter I. Winter II in Brescia was slightly colder, thus experiencing a major temperature incursion, and the PM_0.5_ content was approximately 16 μg/m^3^.

The growth culture trend was often biphasic: after a noteworthy decrease in cell count at the highest dose, cell growth was newly stimulated and, via short-term treatment, could reach the level of the untreated control. This behaviour indicates that there could exist peculiar transporters (for example, ABC transporters) that are able to sustain the export of PM-associated pollutants and decrease their long-term toxicity. Moreover, PM components could differentially activate/inhibit CYP450 (CYP1A1, CYP1B1, CYP1A2)—crucial in BEAS-2B and A549 cells for bioactivating or detoxifying xenobiotics such as PAHs [28]. For example, CYP1A1 is thought to be able to bioactivate PAH, whereas CYP1A2 could detoxify such xenobiotics [29]. Moreover, the difference in cell sensitivity to treatment between bronchial and alveolar cells could be related to the fact that BEAS-2B and A549 cells do not possess the same amount of CYP1A1 and CYP1A2 (e.g., CYP1A2 mRNA were shown to be almost absent in A549 cells as compared to BEAS-2B, in which they were three times more highly expressed). Furthermore, CYP1B1 is highly activated in A549 cells exposed to PAHs. On the contrary, CYP1A1 was greatly expressed in A549 cells, where the expression level was almost 10 times higher than in BEAS-2B cells [30].

Atmospheric conditions play a fundamental role in regulating the level of chemical pollutants in particulate matter in general and in PM_0.5_ in particular. A chemical analysis of the PM organic extracts described by Bonetta and coworkers [19] demonstrated that during spring, a significant decrease in PAH and nitro-PAH concentrations in PM_0.5_ was reported in all cities. Despite not knowing which specific component drives the whole biological effect, Bonetta et al. demonstrated that significant concentrations of IPA (total IPA, Benzo(a)pyrene, Cancerogenic IPA) are present in PM_0.5_ during winter in Brescia and Turin. In addition, concerning Brescia, even if the trend is similar between winter I and winter II, biological effects are quantitatively different and could thus be explained, at least in part, by differences in PAHs and nitroPAHs observed in these two samples [19]. It is thus necessary to consider not only the PM concentration but also the physical and chemical characteristics.

Our attention was directed to tumour promoters since, considering the evidence of an increased risk of lung cancer associated with increasing levels of air pollution, outdoor air pollution and relative PM were classified by the International Agency for Research on Cancer (Lyon, France) as carcinogenic to humans (group1). Intercellular communication via GJ plays an essential role in regulating growth, differentiation, and physiological cell functions, as well as in maintaining tissue homeostasis. The decrease in the extent of this type of communication between cells is an event often associated with carcinogenesis and particularly with the action of tumour promoters such as the TPA ester (12-O-tetradecanoylforbol-13-acetate) [31,32,33]. However, analysing the tumour promotion, we observed that all air samples contained substances that altered the capacity of GJIC, regardless of the season. The adverse effects of PM on human health are notably due in part to PAHs. Bauer et al. [34] showed that PAHs can also act as cocarcinogens in combination with B[a]P. In the Brescia samples, as determined by Bonetta et al. [19], PAHs and B(a)P concentrations were as follows: (i) for winter I, 7.14 and 074 ng/m^3^, (ii) for spring, 0.44 and 0.01 ng/m^3^, and (iii) for winter II, 6.63 and 0.62 ng/m^3^, respectively. However, in our experimental conditions, the GJIC decrease in IAR203 cells was not correlated to PAHs and B(a)P concentrations since inhibition was more marked for winter II > spring > winter I, suggesting that other chemical compounds present in the extracts could be involved. PM_0.5_ concentrations (winter I 12.6, spring 11.8, and winter II 16.0 mg/m^3^) were more related to the GJIC inhibition trend.

Ultimately, the redox status of pulmonary cells was assessed because PM is known to generate oxidative stress. In our experimental conditions, ROS levels remained almost unchanged. This unaffected level of ROS could be due to cells being treated at a low dose (1 m^3^Eq) for 6 h. This dose corresponds to about 37.3 μg, 41 μg, and 55 μg of PM_10_ for winter I, spring, and winter II samples, respectively (or 12.6 μg, 11.8 μg, and 16 μg PM_0.5_ for winter I, spring, and winter II samples, respectively). The results shown in the literature follow experiments performed with about 100 μg/mL PM_10_, thus representing 3.8- to 2.2-times-higher doses. It was also proposed that ROS could be activated either in the short term or the long term, according to the organic chemicals involved. However, examining the GSH/GSSG ratio, we can see that glutathione exists mainly under the oxidised form (GSSG), which could suggest that glutathione exerts its balancing ability to protect cells against oxidative stress. Although PM is known to act on mitochondria, cell viability was assessed via an MTT assay, and non-significant modulation was observed, suggesting that in our experimental conditions these organelles are not the main target of PM_0.5_. The minimal variations in ROS levels vs. GSH contents could be explained by an efficient regulation of glutathione contents mediated by the different enzymatic systems involved in glutathione synthesis/catabolism, as well as protease activation and the modulation of gene expression [35,36]. Moreover, it could also be explained by a delayed response to stressors in ROS synthesis and the regulation of the glutathione balance.

The down-regulation of MMP9 and MMP2 through PM treatment at the higher dose (7.5 m^3^Eq) can be linked to the low level of redox activity. This is a surprising result since MMP activity usually increases during inflammatory processes and in the presence of diesel exhaust, for example [37,38]. On the contrary, our situation seems more similar to the results of Doornaert et al. [39], who demonstrated the inhibition of MMP1. Our results are also closer to experiments performed on rat lungs, as documented by Wei Yi Su and collaborators [40], which showed a time-related decrease in gelatinase mRNA (mainly MMP9) in response to combustion PM. Moreover, 3 h of exposure to ambient PM did not significantly interfere with MMP2 and MMP9 mRNA levels, whatever the PM size fraction considered (<1.7; 1.7–3.7; 3.7–10 μm). MMPs are fundamental proteolytic enzymes involved in lung tissue repair since their activity contributes to the extracellular matrix remodelling process [41]. However, excessive proteolytic activity can result in inflammation and tissue damage (idiopathic pulmonary fibrosis, COVID-19, cancer, etc.) [42,43,44,45]. MMP expression and activity are thus finely regulated by their specific tissue inhibitors in healthy tissues and dysregulated in a pathological environment [46]. However, lung tissue is of course more complex than our in vitro 2D conditions. In our experimental context, the reduction in MMP activity could result from the decreased redox/inflammatory processes related to the initial presence of PM. In this case, a 3D heterotypic cell model will also be essential to better understand the role of these different cell types in regulating tissue homeostasis/dysfunction.

In this multiparametric approach, we saw that PM_0.5_ induced biological responses differently from what was reported in the literature with the PM_10_ fraction. In our culture conditions (i.e., low dose of PM, as compared to the literature), we did not observe early ROS induction, but the biological effect was high enough to activate the GSH balance. We found that 6 hrs of exposure to PM_0.5_ was sufficient to inhibit GJIC, suggesting the tumour promoter potential of PM_0.5_ in lung epithelial cells. Acute and chronic toxicity studies confirmed that PM_0.5_ is differentially cytotoxic according to the season, weather, and geographical localisation. Ultimately, it seems that biotransformation machinery (CYP (cytochromes), ABC transporters, etc.) could play a fundamental role in regulating the PM_0.5_ impact on lung homeostasis.

Our results could also suggest that epithelial cells are not the unique target of PM_0.5_ and that polymorphonuclear (PMN) and/or macrophage activation could play a role in the chronic response to air pollution. The development of three-dimensional (3D) in vitro models consisting of immune cells together with lung epithelial cells could help us better understand the dynamics of the inflammatory response to PM_0.5_.

## 4. Materials and Methods

The MAPEC_LIFE project, funded by the EU Life+ Programme (LIFE12 ENV/IT/000614), the European Union’s environment fund (https://webgate.ec.europa.eu/life/publicWebsite/project/LIFE12-ENV-IT-000614/monitoring-air-pollution-effects-on-children-for-supporting-public-health-policy, accessed on 20 June 2025) includes several partners (the Universities of Brescia, Perugia, Pisa, Salento, and Turin; the Municipality of Brescia; and the CSMT of Brescia). This multicentre Italian cohort study aimed to evaluate the associations between air pollution and early biological effects in 6–8-year-old Italian children (see details of the study design in [47]). Briefly, the oral mucosa cells of 1149 children were collected to evaluate the frequency of micronuclei (MN) and DNA damage. Children were recruited from primary schools in five Italian towns with different levels of air pollution. To evaluate children’s exposure to urban air pollution, PM_0.5_ was collected near each school on the same days as the biological samplings were performed, and was then analysed for its toxic and genotoxic potential. The results on subject characteristics, human health risk, frequency of MN, and in vitro, genotoxic effects of PM have already been published [19,48,49,50].

Collection of particles: High-volume air samplers for collecting atmospheric particulates were used to withdraw and concentrate the ultra-fine particulate samples (PM_0.5_) on glass fibreglass filters. The size of the glass fibre filters was 20.3 × 25.4 cm (8 × 10 inch). Protocols had already published elsewhere [19]. Briefly, after the gravimetric determination of the PM_0.5_ mass, the samples were processed with organic extraction, and then suspended in DMSO to obtain a final concentration equivalent to 1 m^3^/mL. The results were expressed in m^3^Equivalent. Sampling campaigns were performed in 5 cities (Brescia, Pisa, Lecce, Perugia, Turin) over a continuous period of 48 h (thus including day and night). The samples were collected during winter I (November to March) and late spring (April/June). For Brescia, an additional sampling was performed during winter II (November to January). Cells were treated with increasing extract dosage (1 m^3^Eq, 2.5 m^3^Eq, 5 m^3^Eq, and 7.5 m^3^Eq). The studies were performed with these doses based on the volume of air currently assumed during daily breathing (8 to 12 m^3^/d) and were undoubtedly soluble in the culture medium without inducing extreme cytotoxicity due to the solvent.

All chemicals used in this research were purchased, unless otherwise specified, from Sigma-Aldrich (Milan, Italy) and reagents for cell culture from Life Technologies (Monza, Italy). Plasticware for cell cultures was acquired from Falcon unless otherwise specified.

Cell culture: To determine cell tropism, the toxicity studies were performed using two cell lines of human origin A549, recognised as reference models for the in vitro study of the respiratory system. The first cell line, A549 alveolar epithelial cells (Interlab Cell Line Collection, Genova, Italy), expresses many typical features of type II alveolar lung epithelial cells [51]. The second cell line, BEAS-2B bronchial epithelial cells (Sigma Aldrich, Milan, Italy, #95102433), retains the ability to differentiate into squamous cells in the presence of serum; moreover, they are often used in the context of screening studies of chemical and biological agents known to alter the differentiation processes and/or to induce carcinogenesis. Before use, all cell lines were characterised for the absence of mycoplasms (DAPI and RT-PCR). Both cell lines were maintained in DMEM (Dulbecco’s Modified Eagle Medium) medium supplemented with 10% FBS (bovine foetal serum). Cells were seeded at a density of 50,000 cells/cm^2^. The cell growth study was performed in 24-well plates. Cells were subjected to four treatment doses in addition to the untreated control (0 m^3^Eq), namely 1 m^3^Eq, 2.5 m^3^Eq, 5 m^3^Eq, and 7.5 m^3^Eq; each treatment was performed in triplicate and lasted for three days.

- Baseline toxicity study.

Cytotoxic/cytostatic studies of PM_0.5_: To assess the toxic effect of organic samples, growth curves were elaborated after a daily cell count. To this aim, cells were seeded at a density of 50,000 cells/cm^2^ in 24-well plates. The culture medium was eliminated 24 h after seeding, and both cell types were treated with increasing extract dosage (1 m^3^Eq, 2.5 m^3^Eq, 5 m^3^Eq, and 7.5 m^3^Eq), as previously mentioned. Later, after 24 h and every day, the cells were automatically counted using an automated cell counter (TC20 instrument, Biorad, Segrate, Italy).

Study of cell viability via colourimetric assays: A further study performed was the evaluation of cell viability through neutral red (NR) and MTT (tetrazolium salt (3-(4,5-dimethylthiazol-2-yl)-2,5-diphenyltetrazolium bromide) assays, which allowed us to study the ability of cells to retain a dye in their cytoplasm and their metabolic activity, respectively. Both cell lines were seeded at a density of 20,000 cells/cm^2^ in 96-well plates. Experiments were performed in triplicate, after 24 and 48 h of treatment.

MTT colourimetric assay: After the exposition to organic samples, the culture medium was eliminated from the wells, and a solution of MTT 0.2 mg/mL in Hank’s/Hepes buffer was added. After 2 h in the incubator at 37 °C (5% CO_2_), the remaining MTT solution was removed, and an isopropanol acid solution (0.4 M HCl in isopropanol) was added to dissolve formazan crystals. The staining intensity was evaluated by using the SUNRISE spectrophotometric plate reader (Tecan, Cernusco sul, Naviglio, Italy) at a wavelength of 620 nm.

NR Colourimetric assay: The NR solution was prepared the day before the test by diluting (1:80) a concentrated NR solution in DMEM medium with 5% FBS and leaving it overnight in an incubator at 37 °C. This solution was centrifuged twice at 3500 rpm for 10 min at room temperature and then placed in contact with the cells for 3 h at 37 °C (5% of CO_2_). Afterwards, this solution was removed, and the cells were subsequently fixed with a solution of formol-calcium for 1 min and lysed in acetic acid–ethanol. The intensity of the staining was determined after 5 min of stirring, with a microplate reader at a wavelength of 540 nm.

- “Specific endpoints” for toxicity studies.

Study of GJIC through the scrape loading technique:

To evaluate the intercellular communication mediated by gap junctions (GJIC), the scrape loading technique was used in subconfluent cells grown in monolayers, which were injured with a scalpel in the presence of Lucifer Yellow. The tracer trapped inside the cytoplasm can spread to other adjacent cells only if they communicate via gap junction channels. In detail, the A549 cells were seeded at a density of 100,000 cells/cm^2^ in 24-well plates. After 24 h, the cells were treated with the subtoxic 1 m^3^Eq dose for 6 h. Thereafter, the cells were washed 2 times with PBS containing Ca^2+^ and Mg^2+^, and the cell monolayer was delicately cut through a scalpel. The Lucifer Yellow solution was immediately added and incubated for 7 min at 37 °C. After two washes with PBS, the cells were fixed for 5 min with formaldehyde at 4% and subsequently washed with PBS. Cells were rapidly observed under fluorescence microscopy (Olympus, Segrate, Italy). The IAR203 cell line (kindly provided by Paule Martel, INRAE, the National Research Institute for Agriculture, Food and Environment, France) derived from rat liver was also used to perform such an investigation because it has a high communicating potential. The experiments were performed in duplicate. Communicating cells were counted on microphotographs from different areas of the treated cells.

Study of oxidative stress by determining the content of glutathione (GSH) and reactive oxygen species (ROS).

The quantification of ROS and GSH were performed using two kits from Promega (Milan, Italy) ROS-Glo™ H_2_O_2_ and GSH/GSSG-Glo™ Assays, respectively. Both kits use the high-sensitivity properties of bioluminescence. The dosages were determined following Promega’s instructions.

GSH/GSSG assay: This assay uses luciferin-NT, which is converted into luciferin by a glutathione S-transferase enzyme coupled to a firefly luciferase reaction. The light emitted by the reaction is dependent on the luciferin produced, which is proportional to the amount of GSH.

Briefly, the solutions containing the samples and the total glutathione lysis reagent were placed in the wells of suitable 96-well luminometer plates that were subjected to gentle stirring for 5 min and then left to incubate for 30 min in the dark. Luciferin generation reagent was then added to each well and again after 30 min of incubation. By the end, each well received the luciferin detection reagent, which is a luciferase formulation that creates light proportionally to the amount of luciferin produced from Luciferin-NT; finally, the plate was incubated in the dark for another 15 min and, after a short stirring, the reading is performed at the luminometer, which measures the amount of light produced in RLU (Relative Light Units).

ROS assay: This assay, from Promega (Milan, Italy), is based on the use of a peculiar H_2_O_2_ substrate that reacts directly with H_2_O_2_ to generate a luciferin precursor. The precursor, in the presence of Ultra-Glo^TM^ Recombinant Luciferase and d-Cysteine, is converted into luciferin, which reacts with the Luciferase to generate a bioluminescent signal proportional to H_2_O_2_ concentration.

A solution containing luciferin detection reagent (1 mL), D-Cysteine (10 μL), and signal enhancer solution (10 μL) was added to each sample in the 1:1 ratio (*v*:*v*). The mix was deposited in the wells of a suitable 96-well luminometer plate and subsequently incubated in the dark at room temperature for 20 min. Finally, the quantification of the resulting bioluminescence of the photons emitted by the transformation of luciferin, proportional to the concentration of H_2_O_2_, was performed with a luminometer (Berthold Technologies, Arcore, Italy), which expresses the results in RLU (Relative Light Units).

Study of the metalloprotease (MMP) activities involved in the remodelling of the pulmonary extracellular matrix.

Zymography was used to evidence MMP activities; this technique, which facilitates the separation of proteins according to their molecular weight, is based on the electrophoretic migration of proteins on acrylamide/bisacrylamide gel mixed with gelatine, a substrate for MMPs. This way, the different MMPs and their relative isoforms able to digest gelatin can be evidenced (MMP9, MMP2, etc.). Acrylamide/bis acrylamide 10% gels were prepared according to the classical SDS-PAGE method in the presence of 0.1% gelatine. Culture media were collected after treatments with the entire set of concentrations (untreated control; 1, 2.5, 5, 7.5 m^3^Eq) for 24, 48, 72, and 144 h. An amount of 50 μL of the culture media was loaded into each well of the gels. After electrophoretic migration, gels were washed in 2.5% Triton X100 and thereafter in 100 mM Tris/HCl pH 7.4. Finally, the gels were incubated at 37 °C for 48 h in Tris/HCl 100 mM pH 7.4 supplemented with CaCl_2_ and ZnCl_2_ cofactors. The gels were then stained for 30 min with Coomassie Blue R-250 to colour proteins and bleached (2 destain solutions containing acetic acid, methanol, and H_2_O milliQ) to evidence white digested bands (MMP activity) at their specific molecular weights.

### Statistical Analysis

The results are expressed as mean ± SD values from measurements performed in triplicate. Student’s *t*-tests were used to compare treated groups vs. the untreated control. Differences were considered significantly different for a *p*-value < 0.05.

## Figures and Tables

**Table 1:** Determination of IC50 in alveolar (A549) and bronchial (BEAS-2B) cells after 24 h or 48 h of treatment with Brescia-derived samples. IC50 was empirically determined on semi-log curves obtained from data used to study cell growth/toxicity (which were obtained in experiments performed in triplicate). Data are expressed as m^3^Eq. nd: not determinable (e.g., overly low or no toxicity).

Brescia		IC50 at 24 h (m^3^Eq)	IC50 at 48 h (m^3^Eq)
A549	Winter I	nd	nd
	Spring	nd	nd
	Winter II	6.1	nd
BEAS-2B	Winter I	6.3	nd
	Spring	nd	nd
	Winter II	6.7	6.3

**Table 2:** Quantification of communicating cells. After cutting the cell monolayer and adding Lucifer Yellow, only cells communicating via gap junctions were stained and microphotographed. The extension of dye transfer was evaluated in more than 10 different photographic fields for each treatment. Results are expressed as the average of communicating cells ± SEM.

Communicating Cells	Untreated Control	Winter I	Spring	Winter II
A549	1.94 ± 0.08	1.53 ± 0.07	1.56 ± 0.05	1.48 ± 0.07
IAR203	3.60 ± 0.08	2.39 ± 0.07	2.28 ± 0.07	2.13 ± 0.07

**Table 3:** Quantification of the previous zymograms. Quantification was performed using the ImageJ 1.54g software program. All gels were analysed in duplicate. Results present the density of each band corresponding to the 92 kDa (MMP9) and 72 kDa (MMP2) bands and SEM. ND: not determinable.

**Alveolar Cells**		**Winter I**				**Spring**				**Winter II**			
		**MMP9**	**SEM**	**MMP2**	**SEM**	**MMP9**	**SEM**	**MMP2**	**SEM**	**MMP9**	**SEM**	**MMP2**	**SEM**
24 h	0	24,992,882	3,478,117	22,831,460	3,306,950	20,504,128	2,242,435	19,764,231	525,768	15,411,420	217,354	14,877,338	1,447,950
	1	28,849,746	4,120,032	21,705,531	1,593,586	21,884,018	589,597	18,792,141	282,707	14,770,667	91,107	12,440,435	1,525,147
	2.5	28,062,871	1,249,571	20,428,692	1,091,354	20,981,947	17,354	19,638,256	228,500	14,565,091	332,732	14,838,217	690,243
	5	ND	ND	ND	ND	20,919,896	537,182	19,331,493	3707	13,165,728	1,989,440	10,706,192	3,005,490
	7.5	ND	ND	ND	ND	19,132,629	288,329	17,396,775	378,232	10,947,142	1,471,511	10,604,702	117,828
48 h	0	27,475,386	412,621	20,615,409	34,707	20,918,745	2,589,617	17,924,616	3,272,730	13,348,556	372,389	12,304,267	296,414
	1	30,128,336	363,622	20,823,606	604,511	22,152,185	1,955,057	18,073,989	1,940,718	17,506,142	11,147	16,702,595	208,500
	2.5	30,270,033	352,561	37,323,229	16,300,134	25,820,342	1,467,142	20,438,593	970,406	16,340,081	19,207	16,667,338	1000
	5	29,212,472	136,829	20,490,181	507,157	21,486,599	1,123,136	18,499,771	667,742	11,038,803	2000	13,863,581	143,707
	7.5	25,294,462	324,304	19,231,560	901,364	14,307,704	371,889	16,556,301	223,535	6,883,843	23,061	9,024,393	82,561
72 h	0	34,045,469	910,136	50,730,604	1,818,718	16,997,421	1,557,000	13,944,951	596,106	9,801,408	498,768	16,794,970	596,440
	1	36,520,070	405,415	52,627,553	1,364,596	18,188,704	274,647	16,525,407	836,399	13,024,382	407,500	19,399,495	400,500
	2.5	36,543,277	342,086	56,142,897	355,061	15,771,608	1,340,965	16,947,093	570,542	11,571,499	90,232	14,378,424	3,221,178
	5	33,446,852	390,096	71,120,793	385,540	15,665,426	954,904	16,608,124	102,439	11,610,343	342,097	18,744,505	517,753
	7.5	32,219,170	148,757	58,877,792	8,099,421	14,570,047	339,718	14,684,022	294,914	9,539,271	315,389	15,822,863	1,001,253
144 h	0	20,897,901	2,700,835	50,755,629	513,743	15,476,259	207,293	22,572,296	17,146	8,778,232	923,036	21,473,758	782,500
	1	23,593,244	8,805,785	54,543,497	490,854	18,523,183	200,975	23,657,382	155,403	10,378,424	836,329	23,984,804	1,113,546
	2.5	21,394,840	5,447,431	54,543,497	1,144,201	18,535,926	394,940	24,909,796	159,596	11,160,292	123,561	22,967,330	176,243
	5	22,550,876	8,127,396	51,753,187	2,579,486	17,378,194	1,044,915	23,757,725	212,353	10,744,818	1,207,622	23,500,077	1,354,597
	7.5	22,375,426	10,166,138	51,193,987	4,498,193	15,707,662	464,768	20,517,386	441,571	10,081,171	865,682	19,528,773	854,243
**Bronchial Cells**		**Winter I**				**Spring**				**Winter II**			
		**MMP9**	**SEM**	**MMP2**	**SEM**	**MMP9**	**SEM**	**MMP2**	**SEM**	**MMP9**	**SEM**	**MMP2**	**SEM**
24 h	0	18,810,744	1,881,728	19,015,284	3,469,703	19,924,892	496,571	28,916,771	1,015,500	15,249,667	1,481,622	18,072,445	1,727,864
	1	18,982,997	1,557,354	17,584,773	3,723,678	20,217,988	1,568,910	31,383,777	853,293	16,890,545	246,571	20,297,405	1,528,339
	2.5	17,988,997	1,413,596	16,781,284	3,237,532	19,512,210	435,939	31,843,863	1,472,986	17,609,545	1,831,278	20,636,051	2,086,571
	5	16,570,486	1,652,157	16,512,152	2,991,349	19,549,443	1,811,607	33,131,499	906,743	17,017,278	1,498,304	20,816,627	2,339,631
	7.5	13,326,152	441,965	15,718,359	3,109,556	21,151,589	556,268	30,511,817	2,606,375	13,624,106	1,792,718	19,436,713	2,285,253
48 h	0	14,921,137	266,000	17,949,759	467,258	32,853,959	695,768	33,256,417	109,732	16,990,637	1,834,985	17,699,924	2,377,293
	1	15,688,350	23,728	19,426,855	1,794,546	32,075,201	154,818	35,476,720	317,207	19,598,212	1,340,682	19,911,738	1,480,449
	2.5	15,127,012	213,904	17,554,213	1,050,975	29,968,691	1,199,672	33,886,453	254,354	18,743,369	1,621,182	18,657,945	1,949,071
	5	17,564,576	1,089,247	16,611,556	1,154,682	25,090,408	2,255,318	32,135,750	849,743	13,918,167	1,376,486	16,810,313	1,842,581
	7.5	14,646,405	106,975	15,427,303	1,483,400	25,126,310	2,450,190	29,179,715	399,172	11,961,389	1,208,950	17,414,202	1,853,157
72 h	0	13,203,662	217,525	22,668,800	1,075,219	19,270,714	327,000	25,596,746	473,940	25,111,394	2,241,132	22,128,269	164,647
	1	15,153,648	256,147	22,868,786	1,567,356	19,330,778	772,350	24,647,940	3,715,711	25,699,394	968,718	25,954,725	222,404
	2.5	13,574,123	514,036	22,411,189	1,280,002	22,334,382	515,061	23,030,229	305,121	24,868,066	785,218	23,944,982	192,611
	5	13,060,112	131,854	19,537,947	893,124	22,122,271	380,829	23,289,972	720,622	26,827,526	689,708	22,858,396	257,025
	7.5	13,938,187	587,000	17,195,401	1,023,356	19,650,978	1,011,707	23,316,422	720,536	21,779,398	1,358,207	19,895,760	427,318
144 h	0	22,888,453	1,150,940	27,375,486	594,011	20,855,139	2,517,596	26,585,240	60,526	18,904,266	780,389	25,883,645	2,231,546
	1	24,609,231	198,475	27,375,486	584,339	19,792,032	985,096	24,026,936	388,121	19,820,869	813,071	24,238,080	712,525
	2.5	25,143,574	930,718	28,499,078	107,389	21,461,361	83,354	23,041,754	419,768	18,924,327	722,036	22,640,620	522,965
	5	22,357,871	327,914	26,807,228	71,654	23,391,346	1,082,975	21,337,073	23,329	18,036,093	1,090,400	21,006,474	884,597
	7.5	19,929,033	1,311,440	22,993,322	515,157	23,359,589	669,389	18,750,551	39,293	16,966,351	240,536	16,467,538	1,190,117

## Data Availability

Data are held on our laboratory’s external storage disk.

## Appendix A

MAPEC_LIFE Study Group: University of Brescia: Elisabetta Ceretti, Loredana Covolo, Francesco Donato, Donatella Feretti, Andrea Festa, Rosa Maria Limina, Gaia Claudia Viviana Viola, Claudia Zani, Ilaria Zerbini; University of Perugia: Cristina Fatigoni, Sara Levorato, Silvano Monarca, Tania Salvatori, Samuele Vannini; University of Torino: Bonetta Silvia, Giorgio Gilli, Marta Gea, Cristina Pignata, Valeria Romanazzi, Tiziana Schilirò; University of Pisa: Beatrice Bruni, Beatrice Casini, Gabriele Donzelli, Giacomo Palomba; University of Salento: Francesco Bagordo, Antonella De Donno, Mattia De Giorgi, Marcello Guido, Adele Idolo, Alessandra Panico; Comune di Brescia: Camilla Furia; CSMT Gestione S.c.a.r.l.: Paolo Colombi, Roberta Codenotti, Stefano Crottini, Laura Gaffurini, Licia Zagni.
